# Two highly similar DEAD box proteins, OsRH2 and OsRH34, homologous to eukaryotic initiation factor 4AIII, play roles of the exon junction complex in regulating growth and development in rice

**DOI:** 10.1186/s12870-016-0769-5

**Published:** 2016-04-12

**Authors:** Chun-Kai Huang, Yi-Syuan Sie, Yu-Fu Chen, Tian-Sheng Huang, Chung-An Lu

**Affiliations:** Department of Life Sciences, National Central University, Jhongli District, Taoyuan City 32001 Taiwan (ROC)

**Keywords:** DEAD box RNA helicase, Eukaryotic initiation factor 4AIII (eIF4AIII), Exon junction complex (EJC), Rice (*Oryza sativa*)

## Abstract

**Background:**

The exon junction complex (EJC), which contains four core components, eukaryotic initiation factor 4AIII (eIF4AIII), MAGO/NASHI (MAGO), Y14/Tsunagi/RNA-binding protein 8A, and Barentsz/Metastatic lymph node 51, is formed in both nucleus and cytoplasm, and plays important roles in gene expression. Genes encoding core EJC components have been found in plants, including rice. Currently, the functional characterizations of MAGO and Y14 homologs have been demonstrated in rice. However, it is still unknown whether eIF4AIII is essential for the functional EJC in rice.

**Results:**

This study investigated two DEAD box RNA helicases, OsRH2 and OsRH34, which are homologous to eIF4AIII, in rice. Amino acid sequence analysis indicated that OsRH2 and OsRH34 had 99 % identity and 100 % similarity, and their gene expression patterns were similar in various rice tissues, but the level of *OsRH2* mRNA was about 58-fold higher than that of *OsRH34* mRNA in seedlings. From bimolecular fluorescence complementation results, OsRH2 and OsRH34 interacted physically with OsMAGO1 and OsY14b, respectively, which indicated that both of OsRH2 and OsRH34 were core components of the EJC in rice. To study the biological roles of OsRH2 and OsRH34 in rice, transgenic rice plants were generated by RNA interference. The phenotypes of three independent *OsRH2* and *OsRH34* double-knockdown transgenic lines included dwarfism, a short internode distance, reproductive delay, defective embryonic development, and a low seed setting rate. These phenotypes resembled those of mutants with gibberellin-related developmental defects. In addition, the *OsRH2* and *OsRH34* double-knockdown transgenic lines exhibited the accumulation of unspliced rice *UNDEVELOPED TAPETUM 1* mRNA.

**Conclusions:**

Rice contains two eIF4AIII paralogous genes, *OsRH2* and *OsRH34*. The abundance of *OsRH2* mRNA was about 58-fold higher than that of *OsRH34* mRNA in seedlings, suggesting that the OsRH2 is major eIF4AIII in rice. Both OsRH2 and OsRH34 are core components of the EJC, and participate in regulating of plant height, pollen, and seed development in rice.

**Electronic supplementary material:**

The online version of this article (doi:10.1186/s12870-016-0769-5) contains supplementary material, which is available to authorized users.

## Background

The DEAD box RNA helicase family, the largest family of RNA helicases, belongs to helicase superfamily 2. Each DEAD box RNA helicase contains nine conserved amino acid motifs that constitute the helicase core domain. Besides these conserved motifs within DEAD box proteins, there are also N- and C-terminal extension sequences in each DEAD box RNA family member that varies in terms of their length and composition; they have been proposed to provide substrate binding specificity, and to act as signals for subcellular localization or as domains that interact with accessory components [1–3]. DEAD box proteins are found in most prokaryotes and all eukaryotes, including plants [4–10]. Rice is an important staple food crop and is also valuable as a model plant for studies in cereal functional genomics. Although predicted protein sequences in the rice genome database as determined by silico analysis to indicate that there are at least 51 DEAD box proteins in rice [10], the functional characterizations of most of them remain unknown.

Eukaryotic initiation factor 4AIII (eIF4AIII), a DEAD box RNA helicase, is a core component of the exon junction complex (EJC) that also contains MAGO/NASHI (MAGO), Y14/Tsunagi/RNA-binding protein 8A, and Barentsz/Metastatic lymph node 51 [11–16]. The EJC is formed in both the nucleus and the cytoplasm, and plays important roles in gene expression, including the following: (1) It assembles 20–24 bases upstream of each exon of pre-mRNA for its involvement in mRNA splicing [17]. (2) It is involved in nonsense-mediated decay, a surveillance mechanism that degrades mRNA containing premature termination codons [18]. (3) It is involved in the regulation of gene expression at the translational level [19]. (4) It has a role in mRNA subcellular localization [20, 21].

Although most research has been undertaken in mammals, genes encoding core EJC components have been found in plants [22], suggesting that there is structural and functional conservation in the EJC complex among plant and mammalian. However, only limited evidence has been reported on the physiological role of the EJC in plants. In *Arabidopsis*, eIF4AIII interacts with an EJC component, ALY/Ref, and colocalizes with other EJC components, such as Mago, Y14, and RNPS1 [23]. In *O. sativa*, two forms of *MAGO*, *OsMAGO1* and *OsMAGO2*, and two forms of *Y14*, *OsY14a* and *OsY14b*, were analyzed [24–26]. *OsMAGO1* and *OsMAGO2* double-knockdown rice plants displayed dwarfism and abnormal flowers in which the endothecium and tapetum of the stamen were maintained [24]. OsY14b may function in embryogenesis, while the down-regulation of *OsY14b* resulted in a failure to induce plantlets [24]. *OsY14a* knockdown plants also displayed phenotypes similar to those of *OsMAGO1* and *OsMAGO2* double-knockdown rice plants [24]. Moreover, *OsMAGO1* and *OsMAGO2* double-knockdown, and *OsY14a* knockdown transgenic plants showed abnormal accumulation of the pre-mRNA of *UNDEVELOPED TAPETUM 1* (*OsUDT1*), a key regulator of stamen development [24]. These findings indicate that the EJC participates in the regulation of pre-mRNA splicing in rice.

Despite the fact that the functions of homologs of MAGO and Y14 have been demonstrated in rice, it is still unknown whether eIF4AIII is essential for EJC function in rice. In this study, two putative rice DEAD box RNA helicase genes, *OsRH2* (*Os01g0639100*) and *OsRH34* (*Os03g0566800*), were therefore characterized. Both OsRH2 and OsRH34 are homologous to eIF4AIII, which is a member of the eIF4A family, and their gene expression patterns were similar in various rice tissues, but the level of *OsRH2* mRNA was about 58-fold higher than that of *OsRH34* mRNA in seedlings. The results from bimolecular fluorescence complementation (BiFC) analysis showed that both OsRH2 and OsRH34 can interact with OsMAGO1 and OsY14b. Transgenic plants with both *OsRH2* and *OsRH34* knocked down by RNA interference displayed phenotypes that resembled those of mutants with gibberellin-related developmental defects. Moreover, these *OsRH2* and *OsRH34* double-knockdown plants exhibited severe defects in terms of pollen and seed development. The accumulation of *OsUDT1* pre-mRNA was also detected in the *OsRH2* and *OsRH34* double-knockdown transgenic lines. Our data demonstrate that both OsRH2 and OsRH34 are core components of the EJC and play critical roles in regulation of plant height, pollen, and seed development in rice.

## Results

### OsRH2 and OsRH34 are putative DEAD box RNA helicases

**Fig. 1:**
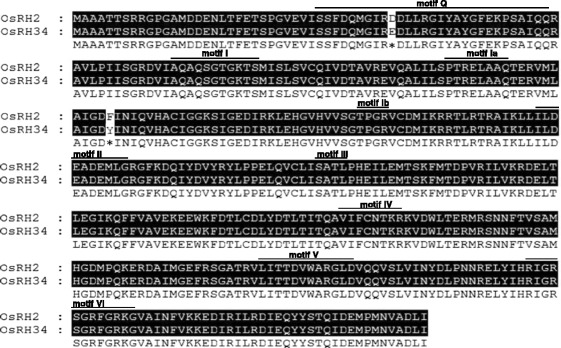
Amino acid sequences and domain structures of the OsRH2 and OsRH34 proteins. A. The amino acid sequences of OsRH2 and OsRH34 were compared using the CLUSTAL W program. Identical amino acid residues are labeled in black. Different amino acid residues are marked by asterisks. The conserved helicase motif is highlighted by a line above it and includes motifs Q, I, Ia, Ib, II, III, IV, V, and VI

**Fig. 2:**
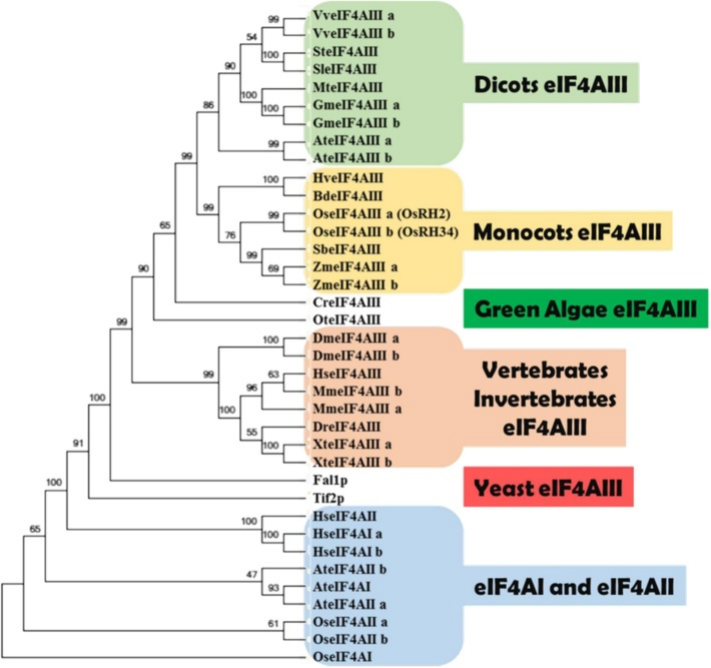
Phylogenetic relationships of eIF4AIII family members. A phylogenetic tree for eIF4AIII in dicots, monocots, green algae, vertebrates, invertebrates, and yeast was generated using MEGA 5. eIF4AIII members from rice, maize, sorghum, and *Brachypodium* are categorized into the monocot group with at least 50 % bootstrap support. Accession numbers of the genes listed here are shown in Additional file 2

To identify rice eIF4AIII homologs, human eIF4AIII protein sequences were used as queries to search protein databases at phytozome and National Center for Biotechnology Information (NCBI). Two eIF4AIII-like putative proteins, encoded by *OsRH2* (*Os01g0639100*) and *OsRH34* (*Os03g0566800*) were identified in rice (Additional file 1). The *OsRH2* is located on rice chromosome 1 and has eight exons. The deduced amino acid sequence of *OsRH2* cDNA consists of nine conserved RNA helicase domains (Fig. 1) and the characteristic amino acid residues D-E-A-D in motif II. Besides, the *OsRH34* gene has eight exons and is located on chromosome 3. The levels of identity between *OsRH2* and *OsRH34* in terms of the DNA sequence and the deduced amino acid sequence were found to be 97 and 99 %, respectively. Phylogenetic relationships were established using amino acid sequences from the eIF4A families of dicots, monocots, green algae, vertebrates, invertebrates, and yeast (Additional file 2), which showed that OsRH2 and OsRH34 are closely related to eIF4AIII and can be clustered into the monocot group (Fig. 2).

### Expression patterns of *OsRH2* and *OsRH34*

**Fig. 3:**
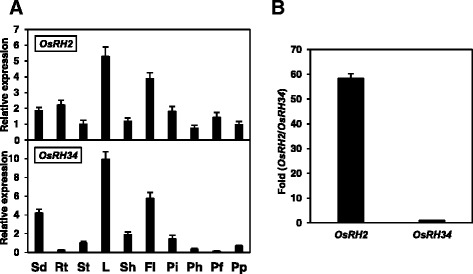
Expression of *OsRH2* and *OsRH34.* **a** qRT-PCR analysis of *OsRH2* and *OsRH34* gene expression in rice. Total RNA was isolated from seedlings (Sd), roots (Rt), stems (St), leaves (L), sheaths (Sh), flag leaves (Fl), booting panicles (Pi), heading panicles (Ph), flowering panicles (Pf), and pollinated panicles (Pp). The rice *Act1* gene was used as an internal control. **b** Absolute quantitative RT-PCR analysis of *OsRH2* and *OsRH34*, in which plasmid DNA was applied as a control to compare the mRNA levels of *OsRH2* and *OsRH34*

To determine the relative expression levels of *OsRH2* and *OsRH34* in rice, total RNA was isolated from a variety of vegetative and reproductive tissues and was subjected to qRT-PCR with specific primers (Additional file 1). The *OsRH2* transcript was expressed in all selected tissues and organs, including roots, stems, leaves, sheaths, panicles, and seedlings (Fig. 3a). Relatively high levels of *OsRH2* mRNA were detected in vegetative leaf blades, flag leaves, and panicles before heading (Fig. 3a). Expression of *OsRH34* was relatively abundant in vegetative leaf blades, flag leaves, and seedlings, whereas its expression was rarely detected in roots, stems, and panicles (Fig. 3a). These results indicate that these two paralogous genes are coexpressed in most selected tissues and organs in rice. To compare the levels of *OsRH2* and *OsRH34* mRNA in rice plants, absolute qRT-PCR was performed. Standard curves were used with a serial dilution of either *OsRH2* cDNA- or *OsRH34* cDNA-containing plasmids. As shown in Fig. 3b, the level of *OsRH2* mRNA was 58-fold higher than that of *OsRH34* mRNA in rice seedlings at the three-leaf stage.

### OsRH2 and OsRH34 were colocalized in nucleus and cytoplasm

**Fig. 4:**
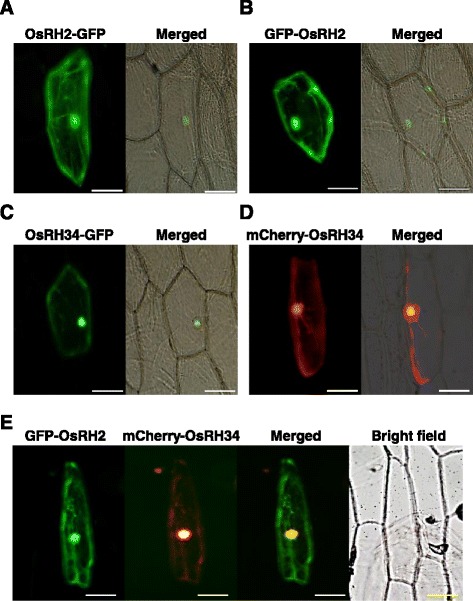
Subcellular localization of OsRH2 and OsRH34. **a** and **b** OsRH2 fluorescence fusion protein was localized in the nucleus and the cytoplasm. Onion epidermal cells were transformed with either 35S::OsRH2–GFP (**a**) or 35S::GFP–OsRH2 (**b**). **c** and **d** Onion epidermal cells were transformed with either 35S::OsRH34–GFP (**c**) or 35S::mCherry–OsRH34 (**d**). **e** Colocalization of GFP–OsRH2 and mCherry–OsRH34 in the nucleus and the cytoplasm. Onion epidermal cells were cotransformed with 35S::GFP–OsRH2 and 35S::mCherry–OsRH34. Bars = 100 μm

To determine the subcellular localization of OsRH2 and OsRH34, plasmids containing an *OsRH2–GFP* fusion gene and *OsRH34–GFP* under the control of the *CaMV 35S* promoter were generated and introduced into onion epidermal cells. Fluorescent signals were emitted from both OsRH2–GFP (Fig. 4a) and OsRH34–GFP (Fig. 4c) in both the nucleus and the cytoplasm. Similar results were obtained in onion cells for the expression of either GFP–OsRH2 (Fig. 4b) or mCherry–OsRH34 (Fig. 4d). To confirm the subcellular localization of OsRH2 and OsRH34, onion cells were cotransformed with GFP–OsRH2 and mCherry–OsRH34. GFP and mCherry signals were colocalized in the nucleus and the cytoplasm (Fig. 4e). These results suggest that the OsRH2 and OsRH34 proteins are localized in both the nucleus and the cytoplasm.

### Both OsRH2 and OsRH34 are components of the EJC core complex

**Fig. 5:**
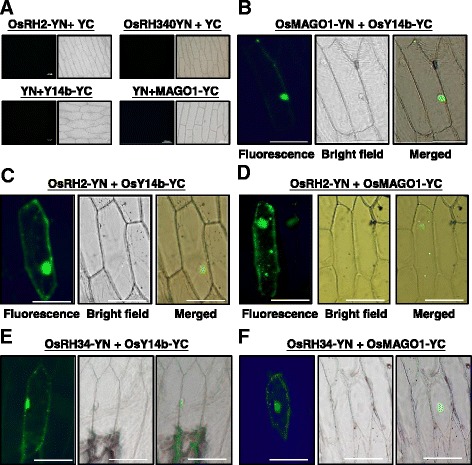
BiFC analysis of the interaction among rice MAGO, Y14, and eIF4AIII in onion epidermal cells. N- and C-terminal fragments of YFP (YN and YC) were fused to the C-terminus of OsRH2, OsRH34, OsMAGO1, and OsY14b, respectively. Onion epidermal cells were cotransformed with combinations of 35S:: OsRH2–YN and 35S::YC, 35S::OsRH34–YN and 35S::YC, 35S::YN and 35S::Y14b–YC, and 35S::YN and 35S::MAGO1–YC as negative controls (**a**) Onion epidermal cells were cotransformed with 35S::OsMAGO1–YN and 35S::OsY14b–YC (**b**), 35S::OsRH2–YN and 35S::OsY14b–YC (**c**), 35S::OsRH2–YN and 35S::OsMAGO1–YC (**d**), 35S::OsRH34–YN and 35S::OsY14b–YC (**e**), 35S::OsRH34–YN and 35S::OsMAGO1–YC. (**e**) Bars = 100 μm

eIF4AIII can interact with Y14 and MAGO to form the EJC core complex in eukaryotic cells [27, 28]. Gong and He [24] have also reported that rice MAGO and Y14 can form heterodimers. To determine whether OsRH2 and OsRH34 were components of the EJC in rice, interactions among rice MAGO, Y14, and eIF4AIII were examined by BiFC. The N-terminus (YN) of yellow fluorescent protein (YFP) was fused at the downstream end of OsRH2 and OsRH34. The C-terminus (YC) of YFP was fused at the downstream end of OsY14b and OsMAGO1. Coexpression of OsRH2-YN and YC, OsRH34-YN and YC, YN and OsMAGO1-YC, YN and OsY14b-YC in onion epidermal cells were used as negative controls for interaction tests among OsRH2, OsMAGO1, and OsY14, and no fluorescent signals were detected (Fig. 5a). The interaction between OsMAGO1 and OsY14b was used as a positive control that exhibited remarkable fluorescent signals in onion cells (Fig. 5b). These two fusion proteins, OsRH2-YN and OsY14b-YC, were coexpressed in onion cells and the YFP fluorescence was observed (Fig. 5c). OsRH2-YN and OsMAGO1-YC coexpressed in onion cells also displayed the YFP signal (Fig. 5d). Meanwhile, YFP fluorescence was also detected upon the coexpression of OsRH34-YN with OsY14b-YC (Fig. 5e) and OsRH34-YN with OsMAGO1-YC (Fig. 5f), respectively. These results indicate that both OsRH2 and OsRH34 directly interact with OsY14b and OsMAGO1, demonstrating that they are indeed a component of the EJC core complex in rice.

The OsRH2 and the OsRH34 were colocalized (Fig. 4), so protein interaction between these two isoforms was further examined by the BiFC analysis. The YFP fluorescent signals were not be observed in onion cells coexpressed with either combinations of OsRH2-YN and OsRH2-YC, OsRH34-YN and OsRH34-YC, OsRH2-YN and OsRH34-YC, or OsRH34-YN and OsRH2-YC (Additional file 3). These results indicated that proteins of OsRH2 and OsRH34 were not able to interact to form homomer or heteromer.

### Characterization of double knockdown of *OsRH2* and *OsRH34* transgenic lines

**Fig. 6:**
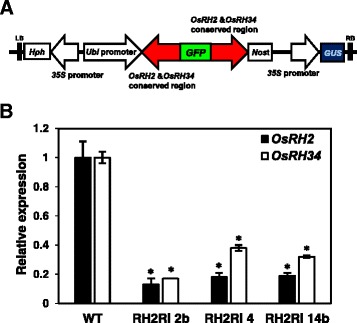
Characterization of *OsRH2* and *OsRH34* double-knockdown transgenic lines. **a** Schematic presentation of the double silencing of *OsRH2* and *OsRH34* of the RNA interference construct. A 271-bp fragment at the 3′ end of *OsRH2* and *OsRH34* conserved region was ligated in sense and antisense orientations to the GFP cDNA and fused downstream of the *Ubi* promoter. **b** Expression of *OsRH2* and *OsRH34* in T1 transgenic rice seedlings. Total RNA was isolated from 14-day-old seedlings and subjected to qRT-PCR using *OsRH2*- and *OsRH34*-specific primers. Rice *Act1* was used as an internal control. Error bars indicate the standard deviations (SD) of triplicate experiments. Gene expression was related to wild-type plants, as 1. * is significantly different from the wild-type plants (Student’s *t* test: *p* <0.05). *OsRH2* and *OsRH34* double-knockdown lines are named as RH2Ri 2b, 4, and 14b. Wild-type line is indicated by WT

To unravel the physiological functions of *OsRH2* and *OsRH34*, a RNA interference mediated genes silencing approach was performed. Because *OsRH2* and *OsRH34* shared extremely high sequence identity, it was difficult to achieve specific gene silencing. Thus, double knockdown of *OsRH2* and *OsRH34* was carried out in rice. To minimize the potential off-target gene silencing, the sequences of 271-bp RNAi designed region at the 3´ end of *OsRH2* cDNA and *OsRH34* cDNA were used as queries to search rice mRNA databases at NCBI. None of region identical of around or more than 16 nucleotides was obtained. Further, a public web-based computational tool developed for identification of potential off-targets, siRNA Scan [29], was applied to search rice mRNA databases, and no potential off-target was detected in the RNAi designed region. Inverted repeat of the 271-bp region was fused at the up- and downstream ends of a GFP coding sequence, and the fusion construct was expressed under the control of the maize ubiquitin gene (*Ubi*) promoter (Fig. 6a) in transgenic rice. Several independent T1 transgenic plants were obtained, and the levels of *OsRH2* mRNA and *OsRH34* were determined by qRT-PCR. As results showed in Fig. 6b, both *OsRH2* mRNA and *OsRH34* mRNA were barely detectable in three independent T1 transgenic lines, RH2Ri 2b, RH2Ri 4, and RH2Ri 14b, indicating that both *OsRH2* and *OsRH34* were knocked down. Therefore, RH2Ri 2b, RH2Ri 4, and RH2Ri 14b lines were selected to address roles of OsRH2 and OsRH34 in rice.

### Reduced plant height in transgenic rice double knockdown of *OsRH2* and *OsRH34*

**Fig. 7:**
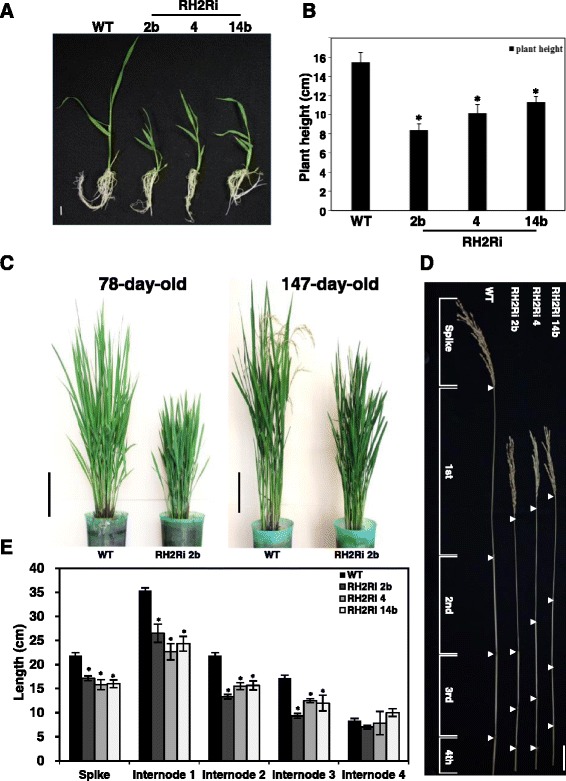
Phenotype of *OsRH2* and *OsRH34* double-knockdown T1 transgenic rice. **a** WT and three independent *OsRH2* and *OsRH34* double-knockdown lines, RH2Ri 2b, RH2Ri 4, and RH2Ri 14b, seedlings were grown on ½ MS agar medium for 10 days and transferred to hydroponic cultures for 7 days. Bar = 1 cm. **b** Quantification of plant height at seedling stages. The plant height of 17-day-old seedlings was measured. Error bars indicate the SD of ten individual plants for each line. * is significantly different from the wild-type plants (Student’s *t* test: *p* <0.05). **c** Comparison of plant height between WT and RH2Ri 2b in 78-day-old plants and 147-day-old plants. Bars = 19 cm. **d** Comparison of internode distance of 4-month-old rice plants among WT, RH2Ri 2b, RH2Ri 4, and RH2Ri 14b. Bars = 5 cm. **e** Determination of internode distance of RH2Ri 2b, RH2Ri 4, RH2Ri 14b, and wild-type plants. Error bars show ± SD (*n* = 20), * is significantly different from the wild-type plants (Student’s *t* test: *p* <0.05)

Significant differences in the height of plants in the T1 transgenic lines were observed. RH2Ri 2b, RH2Ri 4, and RH2Ri 14b showed a dwarf phenotype; their seedlings were 27 to 44 % shorter than those of wild-type plants at 2 weeks old (Fig. 7a and b). Moreover, RH2Ri transgenic plants were shorter than wild-type plants at following growth stage. One example was shown in Fig. 7c, the plant height of RH2Ri 2b T1 plant was 20 and 26 % shorter than wild-type plants at 78-day-old and 147-day-old stages, respectively. Plant height was further compared between wild-type and RH2Ri transgenic plants at the reproductive stage. The culm of wild-type plants contained five internodes, named I to V from top to bottom. Culm lengths of the RH2Ri transgenic plants also appeared to be reduced in each internode region compared with those in the wild-type plants (Fig. 7d and e). The dwarf phenotype of RH2Ri transgenic plants was also observed in a paddy field. Significant differences in plant heights between wild-type plants and RH2Ri plants of the three transgenic T1-T3 generation were observed (Table 1). In addition, the leaves of the RH2Ri transgenic plants were a deeper green and they had a greater number of tillers than the wild-type plants (Fig. 7c).

**Table 1:** Heights (cm) of RH2Ri transgenic plants

	Line			
Generation	WT	RH2i-2b	RH2i-4	RH2i-14b
T1	98 ± 3.2	78 ± 3.0 ^*^	87 ± 2.5 ^*^	89.4 ± 3.8 ^*^
T2	115 ± 4.2	88 ± 5.2 ^*^	95 ± 6.2 ^*^	97 ± 4.1 ^*^
T3	102 ± 5.5	80 ± 2.2 ^*^	85 ± 2.4 ^*^	87 ± 3.7 ^*^

± indicates standard deviation, *n* = 20 for each line

^*^ is significantly different from the wild-type plants (Student’s *t* test: *p* <0.05)

### Severe defects in pollen and seed development in double knockdowns of *OsRH2* and *OsRH34*

**Fig. 8:**
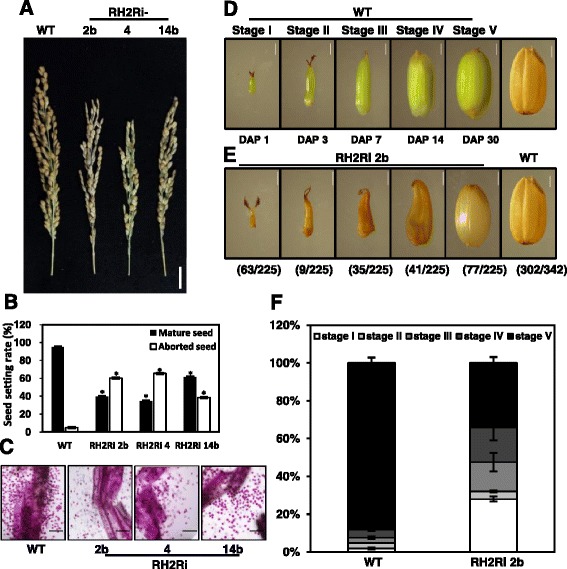
Seed setting rate and seed development in *OsRH2* and *OsRH34* double-knockdown transgenic rice. **a** and **b** A low seed setting rate was observed in *OsRH2* and *OsRH34* double-knockdown plants. **a** Spikelet phenotype of three independent *OsRH2* and *OsRH34* double-knockdown lines, RH2Ri 2b, RH2Ri 4, and RH2Ri 14b. Bars = 5 cm. **b** Determination of the numbers of mature and aborted seeds in *OsRH2* and *OsRH34* double-knockdown lines. Error bars show ± SD (*n* = 20), * is significantly different from the wild-type plants (Student’s *t* test: *p* <0.05). **c** The *OsRH2* and *OsRH34* double-knockdown lines showed defects in pollen development. Bars = 200 μm. **d**–**f** The *OsRH2* and *OsRH34* double-knockdown lines showed defects in embryonic development. **d** Micrographs of husked of wild-type rice seeds at various developmental stages. Rice seeds were harvested at 1, 3, 7, 14, and 30 days after pollination (DAP). **e** The internal seed stages of the RH2Ri 2b line at 30 DAP. **f** Determination of the numbers of the seeds at different stages in the RH2Ri 2b transgenic and wild-type plants

The RH2Ri transgenic plants had 30 ~ 40 % fewer seeds than the wild-type plants (Fig. 8a and b). This marked reduction in the number of seeds suggested that double knockdown of *OsRH2* and *OsRH34* may cause defects in fertilization or seed development. Aborted pollen was previously identified in *OsMAGO1* and *OsMAGO2* double-knockdown plants and *OsY14a* knockdown plants [24]. To address whether OsRH2 and OsRH34 function in the male gametophyte development, pollen viability of RH2Ri transgenic plants was determined by the Alexander staining. In Fig. 8c, aborted pollens were more in RH2Ri transgenic plants than that in wild type, suggesting that double knockdowns of *OsRH2* and *OsRH34* affected male gametophyte development.

On the other hand, the levels of seed development normally seen at 1, 3, 7, 14, and 30 days after pollination (DAP) were set as stage I to stage V, respectively (Fig. 8d). Most seeds in the wild-type plants had developed to stage V at 30 DAP (Fig. 8d). However, in the RH2Ri 2b transgenic plants, the level of seed development at 30 DAP varied, from stage I to V; about one-third of the plants remained at stage I, one-third were at stages II, III, or IV, and one-third formed mature seeds (stage V) (Fig. 8e and f). These phenotypes suggested that the *OsRH2* and *OsRH34* genes play critical roles in the development of rice seeds.

### Exogenous gibberellic acid (GA) partially rescues the phenotype of RH2Ri transgenic plants and double knockdown of *OsRH2* and *OsRH34* influences on GA biosynthesis and GA signaling genes

**Fig. 9:**
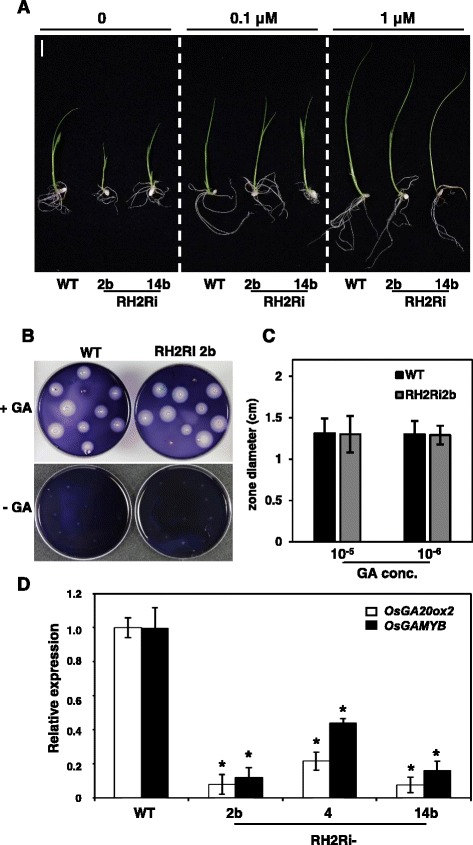
Effect of exogenous GA on the *OsRH2* and *OsRH34* double-knockdown T1 transgenic rice. **a** Three-day-old rice seedlings were incubated in water containing 0, 0.1, and 1 μM GA3 for 7 days. Bars = 1 cm. **b** A starch plate assay of α-amylase activity. Embryoless half seeds were incubated on starch plates with 10^−6^ M GA3 for 2 days. **c** Quantification of clear zone diameter on starch plates with 10^−5^ and 10^−6^ M GA3. Error bars show ± SD (*n* = 40). **d** Expression of *OsGA20ox2* and *OsGAMYB* in the *OsRH2* and *OsRH34* double-knockdown seedlings. Total RNAs were isolated from three-leaf-stage seedling and subjected to qRT-PCR. Rice *Act1* as an internal control. Error bars indicate the SD of four replicate experiments with two biological replicates. Gene expression was related to wild-type plants, as 1. * is significantly different from the wild-type plants (Student’s *t* test: *p* <0.05)

Phenotypes of the OsRH2 RNAi transgenic plants included dwarf, reduced internode length, deep green in the leaf color, increased tiller number, abnormal seed development and reduced seed germination rate, are similar to mutants deficient in GA biosynthesis or GA signaling pathway. To investigate whether OsRH2 and OsRH34 are involved in the GA biosynthesis or signaling pathway, rice seedlings were treated with 0.1 and 1 μM GA3. Elongation of the dwarf phenotype of 10-day-old RH2Ri seedlings was recovered partially by GA3 treatment (Fig. 9a). To further characterize of GA sensing in RH2Ri transgenic plants, starch plate assay for activity of α-amylase from aleurone layer cells was conducted. The embryoless half-seeds were placed on starch plates with or without 1 μM GA3 for 2 days, and then starch plates were stained with iodine. Activity of α-amylase was not detected in RH2Ri 2b and wild-type embryoless half seeds without treatment of GA3 (Fig. 9b). Cleared zone was detected both in GA3 treatment of half seeds, and no difference in cleared zone size was observed between wild-type and RH2Ri 2b transgenic lines (Fig. 9b and c). These results demonstrated that RH2Ri transgenic plants were responsive to exogenously supplied GA3.

To investigate the role of OsRH2 and OsRH34 in GA biosynthesis and GA signaling, the expression levels of the *OsGA20ox2*, a gene encoded for GA biosynthesis, and the *OsGAMYB*, a transcription factor in GA signaling, were determined. Total RNAs were isolated from three-leaf-stage of RH2Ri transgenic seedlings and subjected to qRT-PCR analyses. The mRNA levels of the *OsGA20ox2* and the *OsGAMYB* were significantly decreased in various RH2Ri lines, compared to the wild type (Fig. 9d). This result suggested that OsRH2 and OsRH34 participate the regulation of GA biosynthesis and GA signaling pathways.

### Double knockdown of *OsRH2* and *OsRH34* transgenic plants exhibit accumulation of unspliced *OsUDT1* mRNA

**Fig. 10:**
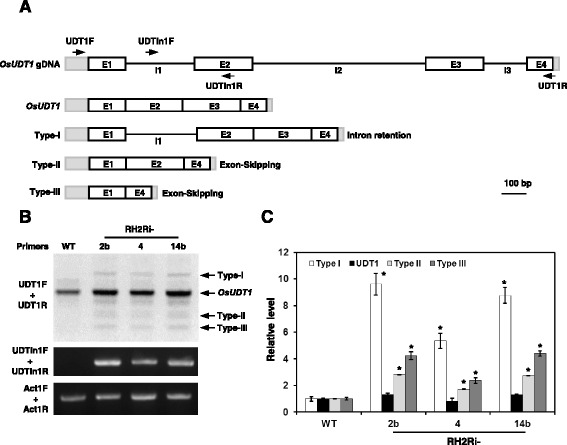
Accumulation of abnormal *OsUDT1* transcripts in *OsRH2* and *OsRH34* double-knockdown plants. **a** Illustration of gene structure and abnormal transcript structures of *OsUDT1* [24], and the positions of primers used for RT-PCR analysis. Gray rectangles, UTRs; white rectangles, exons (E); lines, introns (I). **b** and **c** Accumulation of *OsUDT1* abnormal transcripts. Total RNAs were isolated from inflorescence of WT, RH2Ri 2b, RH2Ri 4, and RH2Ri 14b plants at vegetative stage. Unspliced *OsUDT1* pre-mRNAs were detected by RT-PCR analysis with specific primers (Additional file 1). *Act1* mRNAs were used as internal control. **c** Relative level of abnormal (type I, II and III) and mature *OsUDT1* mRNAs were determined by Image J with normalization relative to the WT. Error bars indicated the SD of four replicate experiments with two biological replicates. Level of DNA fragment was related to wild-type plants, as 1. * is significantly different from the wild-type plants (Student’s *t* test: *p* <0.05)

It has been demonstrated that OsMAGO1, OsMAGO2, and OsY14b are involved in the splicing of *OsUDT1* mRNA [24]. Both OsRH2 and OsRH34 are one component of the EJC core complex, suggesting that double knockdowns of *OsRH2* and *OsRH34* may affect *OsUDT1* mRNA maturation. Total RNA was isolated from inflorescence of plants and subjected to RT-PCR using specific primers (Fig. 10a, Additional file 1) for amplifying fragments of *OsUDT1* mRNA. Four fragments, namely type I, mature, type II, and type III, were amplified using UDT1R and UDT1F primers (Fig. 10b). The accumulated levels of the type I, type II, and type III were higher in three independent *OsRH2* and *OsRH34* double-knockdown lines than wild type (Fig. 10b and c). Using the UDTIn1F and UDTIn1R primer pair to specifically amplify the type I fragments (Fig. 10b), more accumulated unspliced type I *OsUDT1* mRNAs were detected in these three independent *OsRH2* and *OsRH34* double-knockdown transgenic lines, as compared to wild type (Fig. 10b and c). These results indicate that OsRH2 and OsRH34 play critical roles in the accurate splicing of *OsUDT1* pre-mRNA.

## Discussion

In this study, two DEAD box RNA helicase genes, *OsRH2* and *OsRH34*, were characterized in rice. Amino acid sequence analysis indicated that OsRH2 and OsRH34 share 99 % identity and 100 % similarity, suggesting that these two DEAD box RNA helicases might have similar biochemical properties in rice. Both OsRH2 and OsRH34 are homologous to eIF4AIII, which is a member of the eIF4A family. eIF4AIII is a core component of the EJC, which is one of the fundamental factors involved in post-transcriptional processes in eukaryotes [17, 30]. Besides eIF4AIII, the EJC also contains three other subunits, MAGO, Y14, and Btz [28]. The results obtained in the present study demonstrate that both OsRH2 and OsRH34 interact physically with OsMAGO1 and OsY14b. Three independent *OsRH2* and *OsRH34* double-knockdown transgenic lines showed phenotypes that were similar to those of plants in which the *OsY14a* gene had been knocked down or both *OsMAGO1* and *OsMAGO2* had been knocked down, namely, reduced plant height and abnormal endothecium and tapetum in flowers [24]. Thus, OsRH2 and OsRH34 are a core component of the EJC in rice.

Immunofluorescence microscopy indicated that eIF4AIII was localized to the nucleoplasm [28] in HeLa cells; a similar localization pattern of eIF4AIII was observed for transiently expressed myc-eIF4AIII [28]. However, excessive eIF4AIII were found in the cytoplasm by subcellular fractionation analysis [28, 31]. These studies indicated that eIF4AIII is localized in the nucleus and the cytoplasm. In terms of the results of the subcellular localization of the OsRH2–GFP and GFP–OsRH2 fusion protein, fluorescence was detected in the nucleus and the cytoplasm. Similarly, the OsRH34–GFP and mCherry–OsRH34 fluorescence was detected in the nucleus and cytoplasm. These results indicate that both OsRH2 and OsRH34 proteins are localized in the nucleus and the cytoplasm, and also suggest that they can shuttle between these two locations. Indeed, *Arabidopsis* eIF4AIII is mainly localized in the nucleoplasm under normal growth conditions, but is located in the nucleolus and forms splicing speckles under hypoxic stress [23].

The function of the EJC in rice is poorly understood. However, recently, the EJC core subunits OsMAGO1, OsMAGO2, OsY14a, and OsY14b were identified. It has been identified that there are different types of MAGO-Y14 complex, and variation in their specific functions has been proposed [25]. Knockdown of a single *MAGO* did not lead to any visible phenotype, while the double knockdown of *MAGO* genes in rice plants led to dwarfism with abnormal flowers [24], suggesting that *OsMAGO1* and *OsMAGO2* are functionally redundant. The phenotype of *OsY14a* knockdown rice plants matched that of *OsMAGO1* and *OsMAGO2* double-knockdown plants, while the knockdown of *OsY14b* led to failure of the induction of plantlets [24], suggesting the functional specialization of OsY14b in embryogenesis. The amino acid sequences of OsRH2 and OsRH34 were found to be highly conserved, and their gene expression patterns were also similar in various rice tissues. However, the abundance of *OsRH2* mRNA was about 58-fold higher than that of *OsRH34* mRNA in seedlings. These results suggest that OsRH2 and OsRH34 may be functionally redundant, and that OsRH2 plays a major role in rice. Since DNA sequence of *OsRH2* and *OsRH34* are too similar to make specific gene silencing in rice, we therefore cannot rule out a possibility whether each of them has specific functions in rice.

We introduced *OsRH2* mRNA-based interfering RNA into rice and knocked down both the *OsRH2* and the *OsRH34* genes in the same transgenic line. The *OsRH2* and *OsRH34* double-knockdown transgenic lines showed a dwarf phenotype. The number of nodes and the internode distance in *OsRH2* and *OsRH34* double-knockdown lines were less than in the wild type. In addition, the leaves of the *OsRH2* and *OsRH34* double-knockdown transgenic plants were a deeper green and they had a greater number of tillers than the wild-type plants. These phenotypes are similar to those of mutants with defects in gibberellin signaling [32–35] or gibberellin biosynthesis [36–40]. Exogenous GA was able to partially rescue the dwarf phenotype and induce α-amylase activities in aleurone layers of the *OsRH2* and *OsRH34* double-knockdown transgenic lines. The expression levels of the *OsGA20ox2* were decreased in the *OsRH2* and *OsRH34* double-knockdown transgenic lines as compared to WT. These results suggested that the dwarf phenotype of the *OsRH2* and *OsRH34* double-knockdown transgenic lines may due to the decreased level of GA. However, low levels of *OsGAMYB* mRNA were detected in the *OsRH2* and *OsRH34* double-knockdown transgenic lines. In a previous study, *OsY14a* knockdown rice plants and those with double knockdown with *MAGO* genes also exhibited dwarfism with abnormal flowers, and low level of the *OsGA20ox2* and the *OsGAMYB* mRNA [24]. Thus, the function of EJC was suggested to be strongly correlated with the gibberellin action in rice.

There was a discrepancy in the seed setting rate between the wild-type and *OsRH2* and *OsRH34* double-knockdown transgenic lines. The ratio of mature seeds to total seeds in the *OsRH2* and *OsRH34* double-knockdown transgenic lines was lower than in the wild type. The aborted pollen phenotype observed in the *OsRH2* and *OsRH34* double-knockdown plants was consistent with previously identified in *OsMAGO1* and *OsMAGO2* double-knockdown plants and *OsY14a* knockdown plants [24]. These results suggest that the low seed setting rate may be caused by a defective EJC, which affects pollen development. However, it is necessary to address whether the EJC is also involved in female gametophyte development. Alternatively, seed development was compared between the wild type and the *OsRH2* and *OsRH34* double-knockdown transgenic lines. After pollination, 90 % of the seeds of the wild type developed to the mature stage. In contrast, in the transgenic lines, one-third of the seeds developed to maturity, one-third remained at an intermediate stage, and one-third did not progress beyond a very early stage. These results suggested that the double knockdown of *OsRH2* and *OsRH34* impaired seed development. Taking these findings together, the double knockdown of the *OsRH2* and *OsRH34* genes may cause defects in pollen and seed development.

In eukaryotic cells, nonsense-mediated mRNA decay (NMD), a surveillance mechanism, eliminates mRNA that contains nonsense mutations or has acquired premature termination codons because of aberrant splicing [18]. It is thus an effective safeguard for eliminating aberrant gene expression [18, 41–44]. The EJC has been demonstrated to be involved in the post-transcriptional processing of mRNA, including mRNA splicing and NMD, in eukaryotes [45]. In rice, *OsMAGO1* and *OsMAGO2* double-knockdown plants and *OsY14a* knockdown plants exhibited abnormal splicing of *OsUDT1* transcripts. Multiple types of *OsUDT1* mRNA were detected in *OsMAGO1* and *OsMAGO2* double-knockdown plants and *OsY14a* knockdown plants [24]. In the present study, double knockdown of the *OsRH2* and *OsRH34* genes also led to abnormal *OsUDT1* pre-mRNA splicing accumulation. Thus, the knockdown of one component of the EJC causes defects of EJC function, which is strongly correlated to the accumulation of certain abnormal pre-mRNA. However, this type of intron retained pre-mRNA accumulation may be due to an EJC dependent-splicing defect or an EJC dependent-NMD defect. Future studies to identify the proteins that interact with OsRH2 or OsRH34 might provide more information on the specificity of the function of EJC in rice.

## Conclusion

The EJC contains four core components, eukaryotic initiation factor 4AIII (eIF4AIII), MAGO/NASHI, Y14/Tsunagi/RNA-binding protein 8A, and Barentsz/Metastatic lymph node 51, and plays important roles in gene regulation. Genes encoding core EJC components have been found in rice, and currently.the functional characterizations of MAGO and Y14 homologs have been demonstrated in economically important crop, rice. However, little is known about how important of eIF4AIII in rice. In this study, two rice eIF4AIII homologous genes, *OsRH2* and *OsRH34*, were identified. Deduced amino acid sequence of OsRH2 and OsRH34 share 99 % identity and 100 % similarity. Both rice eIF4AIII fluorescent fusion proteins were localized in the cytoplasm and the nucleus. Moreover, OsRH2 and OsRH34 can interact with rice MAGO and Y14, indicating that OsRH2 and OsRH34 are core components of the EJC. OsRH2 and OsRH34 may be functionally redundant, but the abundantly expressed OsRH2 may play a major role in rice. Double-knockdown of *OsRH2* and *OsRH34* exhibited severe defects in terms of plant height, pollen, and seed development. Moreover, double knockdown of the *OsRH2* and *OsRH34* genes led to decrease in expression levels of *OsGA20ox2* and the *OsGAMYB* and abnormal accumulation of *OsUDT1* pre-mRNA. These visible and molecular phenotypes caused by *OsRH2* and *OsRH34* double-knockdown are similar to *OsMAGO1* and *OsMAGO2* double-knockdown plants and *OsY14a* knockdown plants. Collectively, our findings demonstrate the eIF4AIII proteins, OsRH2 and OsRH34, play critical roles in the functional rice EJC.

## Methods

### Plant materials and growth conditions

The rice cultivar *Oryza sativa* L. cv Tainung 67 (TNG67) was collected from the Taiwan Agricultural Research Institute and used in this study. Transgenic rice plants were cultivated at the Agricultural Experiment Station, National Chung-Hsing University (Taichung, Taiwan). For seed germination, seeds were de-hulled, sterilized with 3 % NaOCl for 30 min, and washed extensively with sterile water. Sterilized seeds were placed on ½ Murashige Skoog (MS) agar medium, and then cultivated in a growth chamber at 28 °C under constant light. Seedlings at the three-leaf stage were transferred into hydroponic culture medium (Kimura B solution) for 2 days and then used for various treatments.

### Primers

The nucleotide sequences of all primers used for plasmid construction, PCR, RT-PCR, and qRT-PCR analyses are listed in Additional file 1.

### Plasmids

Plasmid pMDC43 [46] was used for fusion of the OsRH2–GFP chimeric protein. Plasmids pSAT4-DEST-nEYFP-C1 and pSAT5-DEST-cEYFP-C1, were used as gateway vectors for the BiFC assay, and were obtained from the *Arabidopsis* Biological Resource Center. The pCAMBIA vectors were obtained from CAMBIA. 

### Plasmid construction

The *OsRH2* and *OsRH34* coding regions were amplified with specific primers (Additional file 1) by Phusion High-Fidelity DNA Polymerase (NEB, Ipswich, MA, USA) using the cDNA of seedlings at the three-leaf stage as templates. The PCR products were cloned into the yT&A cloning vector (Yeastern, Taipei, Taiwan) to generate pOsRH2 and pOsRH34, respectively. To investigate the subcellular localization of OsRH2 and OsRH34, their full-length cDNA fragments were excised from pOsRH2 and pOsRH34 with *Asc*I and *Not*I, and then ligated into the same sites of pENTR-TOPO vector to generate pOsRH2-ENTR and pOsRH34-ENTR vectors. Using LR clonase (Invitrogen, Carlsbad, CA), recombination was carried out to transfer *OsRH2* and *OsRH34* DNA fragments from entry clones to the destination vector, pMDC43, to generate the GFP-OsRH2 and GFP-OsRH34, respectively. The *OsRH2* and *OsRH34* DNA fragments were also constructed into the pMDC85 to generate the OsRH2-GFP and OsRH34-GFP plasmids, respectively. To construct the OsRH34-mCherry expression vector, a mCherry destination vector, pMDC43m, was generated by replacing the GFP with mCherry in pMDC43. The expression plasmid of mCherry-OsRH34 was generated by LR clonase.

For the BiFC assay, full-length coding regions of OsRH2, OsMAGO1, and OsY14b were amplified with specific primers and then subcloned into the pDonor221 binary vector between the *att*L1 and *att*L2 sites using BP clonase (Invitrogen). Each fragment was subcloned into pSAT4-DEST-nEYFP-C1 and pSAT5-DEST-cEYFP-C1 (B) binary vectors using LR clonase to generate OsRH2–, OsMAGO1–, and OsY14b–YFP (n), and OsRH2–, OsMAGO1–, and OsY14b–YFP (c) fusion genes.

For construction of the *OsRH2* interfering RNA vector, a 271-bp DNA fragment containing 153 bp of the coding region and 117 bp of the 3′’ UTR of *OsRH2* was amplified using specific primers (Additional file 1). This DNA fragment was cloned into the yT&A cloning vector, generating pRH2Ri. Green fluorescent protein (GFP) cDNA was amplified by PCR using a forward primer and a reverse primer (Additional file 1), and was then subcloned into the yT&A cloning vector, generating pGFPRI. The *OsRH2* RNAi DNA fragment was isolated from pRH2Ri by digestion with *Eco*RI and *Bam*HI, the GFP DNA fragment was isolated from pGFPRI by digestion with *Eco*RI, and these two fragments were ligated into the *Bam*HI site of the pAHC18 expression vector, generating pAHC18-OsRH2-Ri. This RNA silencing construct was linearized by digestion with *Hin*dIII and inserted into the *Hin*dIII site of the pCAMBIA1301 binary vector for *Agrobacterium*-mediated gene transformation.

### RT-PCR and qRT-PCR analyses

Total RNA was isolated from whole seedlings and various tissues of mature plants using Trizol reagent (Invitrogen) and then treated with RNase-free DNase I (NEB) to remove genomic DNA contamination. First-strand cDNA was synthesized using RTace reverse transcriptase (Toyobo, Osaka, Japan) with oligo-dT primers. A 20-fold dilution of the resultant first-strand cDNA was subjected to PCR (22–35 reaction cycles) with gene-specific primers (Additional file 1). For the qRT-PCR reaction, first-strand cDNA was synthesized using SuperScript III Reverse Transcriptase (Invitrogen). A 10-fold dilution of the first-strand cDNA was subjected to qRT-PCR using FastStart Essential DNA Green Master (Roche, Basel, Switzerland) and an iQ5 RT-PCR machine (Bio-Rad, Hercules, CA, USA), in accordance with the manufacturers’ instructions. The PCR procedure was independently repeated at least three times. The relative gene expression levels are expressed as ratios of the abundance of the target gene’s mRNA to that of *Act1* mRNA. Data were analyzed using the iQ5 2.1 software provided by the manufacturer. The gene-specific primers used for qRT-PCR are listed in Additional file 1.

### Plant transformation

Rice embryonic calli were induced from germinated seeds on N6 solid medium with 9 μM 2,4-dichlorophenoxy. *Agrobacterium tumefaciens* strain EHA105 was used to perform rice transformation, as previously described [47]. Transformed calli were selected on N6 medium containing 25 mg/L hygromycin B.

### Subcellular localization analysis and BiFC assay

The onion bulb epidermis was prepared and particle bombardment was carried out as described previously [48, 49] with a PDS-1000 biolistic device (Bio-Rad) at 1100 psi. To introduce the plasmid DNA, the bombarded material was cultured in MS medium for 24 h, and then observed and imaged with an Olympus IX71 inverted fluorescence microscope (Olympus, Tokyo, Japan) with a digital camera. The Olympus UMWIBA3 and the Olympus U-MWIGA3 filters were used to obtain GFP and mCherry images, respectively, and images were merged by the DP Manager program.

For BiFC analysis, various combinations of expression vector carriers with YFPN- and YFPC-fused genes were coexpressed in epidermal cells of onion bulb epidermis by particle bombardment. The YFP signal was observed using an Olympus IX71 inverted fluorescence microscope with the Olympus UMWIBA3 filter.

### GA treatment

Sterilized seeds were placed on ½ MS agar medium, and then cultivated in a growth chamber at 28 °C under constant light for 3 days. Seedlings were transferred into hydroponic culture medium (Kimura B solution) with various GA concentrations for 7 days.

### α-amylase activity assay

Embryoless half seeds (endosperms) were sterilized with 3 % NaOCl for 30 min, washed extensively with sterile water. Each plate contained 16 half seeds that were arranged in a small circle. The plates were incubated in the dark for 1–3 days at 30 °C and then stained with iodine solution. The sizes of colorless zone were measured.

### Ethics approval and consent to participate

Not applicable.

### Consent to publish

Not applicable.

### Availability of data and materials

The data sets supporting the results of this article are included within the article and its additional files.

## Additional files

Additional file 1: Primers used in the study. (DOCX 17 kb) Additional file 2: Accession numbers and proteins homologous to eIF4A. (DOCX 18 kb) Additional file 3: BiFC analysis of the interaction between OsRH2 and OsRH34. (PPTX 122 kb)

## Abbreviations

Act: actin
ALY/Ref: Aly/REF export factor
BiFC: bimolecular fluorescence complementation
DAP: days after pollination
eIF4A: eukaryotic initiation factor 4A
eIF4AIII: eukaryotic initiation factor 4AIII
EJC: exon junction complex
GA: gibberellic acid
GA20ox2: gibberellin 20 oxidase 2
GAMYB: GAMYB transcription factor
GFP: green fluorescent protein
MAGO: exon junction complex mago nashi
mCherry: mCherry red fluorescent protein
MS medium: Murashige Skoog medium
NCBI: National Center for Biotechnology Information
NMD: nonsense-mediated mRNA decay
qRT-PCR: quantitative real time reverse transcription polymerase chain reaction
RH: RNA helicase
RNPS1: RNA-binding protein S1
Ubi: maize ubiquitin gene
UDT1: undeveloped tapetum 1
Y14: exon junction complex protein Y14
YFP: yellow fluorescent protein
