# Supplementary material for: Two highly similar DEAD box proteins, OsRH2 and OsRH34, homologous to eukaryotic initiation factor 4AIII, play roles of the exon junction complex in regulating growth and development in rice
Source: BMC Plant Biol. 2016 Apr 12;16:84. doi: 10.1186/s12870-016-0769-5 (PMC4830029; doi:10.1186/s12870-016-0769-5)
Supplement: Additional file 1: — Primers used in the study. (DOCX 17 kb) [file 12870_2016_769_MOESM1_ESM.docx]

| Additional file 1: Primers used in this study | | |
| --- | --- | --- |
| Primer | Sequence | Purpose |
| OsRH2F | AGAGCGGCCGCAATGGCGCGGCCACCACG | OsRH2 full-length cDNA construct & GFP-OsRH2 construction |
| OsRH2R | AGAGGCGCGCCAGACCAGATGATC |  |
| OsRH34F | AGAGCGGCCGCAATGGCGCGGCCACCACG | OsRH34 full-length cDNA construct & GFP-OsRH34 construction |
| OsRH34R | AGAAGGCGCGCCAACCCTTTTTAGGTTCAC |  |
| OsRH2RiF | AACGAATTCTACATCCATCGCATTG | OsRH2 RNAi construction |
| OsRH2RiR | CACGGATCCAACCCTTTAAAGTCC |  |
| NOsRH2F | AGAGCGGCCGCAATGGCGCGGCCACCACG | OsRH2-GFP construction |
| NOsRH2R | TAGGGCGCGCCAATTAGATCAGCAACAT |  |
| attBRH2F | AAAAAGCAGGCTCCACCATGGCGGCGGCCACCACGTCGCGG | OsRH2 BiFC construction |
| attBRH2R | AGAAAGCTGGGTAAATTAGATCAGCAACATT |  |
| attBMAGO1F | AAAAAGCAGGCTCCACCATGGCGACGGGCGGCGCCGCCGGCGA | OsMAGO BiFC construction |
| attBMAGO1R | AGAAAGCTGGGTAAGATTGAATAGGCTTGA |  |
| attBY14bF | AAAAAGCAGGCTCCACCATGGCGGCGGCGGCGAGGACG | OsY14b BiFC construction |
| attBY14bR | AGAAAGCTGGGTACATGTCAAGGCAGCAAGCCTG |  |
| nEYFPF | GACACCCTGGTGAACCGCATCGAGCTGAAG | BiFC construction |
| nEYFPR | CTTCAGCTCGATGCGGTTCACCAGGGTGTC |  |
| cEYFPF | GCTGCTGCCCGACAACCACTACCTGAGCTA | BiFC construction |
| cEYFPR | TAGCTCAGGTAGTGGTTGTCGGGCAGCAG |  |
| RH2RiCF | AGTGTACAGATACCTCCCTC | Real-time PCR |
| RH2RiCR | ATCATAAAGATCACAAAGCGTG |  |
| RH34RiCF | AACGAATTCACAGGCAGGAAGTCTCC | Real-time PCR |
| RH34RiCR | GCGGAGGAGGTCCTCGCGGATC |  |
| GA20ox2F | CATGGAAGGAGACCCTCTCCTT | Real-time PCR and RT-PCR |
| GA20ox2R | TTGCACCGCATGATTGAGCTGCTG |  |
| GAMYBF | ATGTATCGGGTGAAGAGCGAGAG | Real-time PCR and RT-PCR |
| GAMYBR | GCTGGTAGGATAGATGGGTAG |  |
| Act1F | AGACCTTCAACACCCCTGCTA | Real-time PCR and RT-PCR |
| Act1R | CAGGGCGATGTAGGAAAGCTT |  |
| UDT1F | GAGTTTGAGACTTGAGGCTGC | RT-PCR |
| UDT1R | AGTGTCTCAGATGCTTGGAAC |  |
| UDTIn1F | CCACCAGCTGATTCTTGCACT | RT-PCR |
| UDTIn1R | GCAGCTCAAGAACCTCGTTC |  |
| 18SF | GTGACGGGTGACGGAGAATTA | RT-PCR |
| 18SR | ACACTAAAGCGCCCGGTATTG |  |
