# Supplementary material for: Two highly similar DEAD box proteins, OsRH2 and OsRH34, homologous to eukaryotic initiation factor 4AIII, play roles of the exon junction complex in regulating growth and development in rice
Source: BMC Plant Biol. 2016 Apr 12;16:84. doi: 10.1186/s12870-016-0769-5 (PMC4830029; doi:10.1186/s12870-016-0769-5)
Supplement: Additional file 2: — Accession numbers and proteins homologous to eIF4A. (DOCX 18 kb) [file 12870_2016_769_MOESM2_ESM.docx]

| Additional file 2: Accession numbers and proteins homologous to eIF4A | | | | |
| --- | --- | --- | --- | --- |
| Ortholog | Name | Species | Accession Number | Size (aa) |
| Monocots | OseIF4AIII a | *Oryza sativa* | NP_001043673.1 | 404 |
|  | OseIF4AIII b | *Oryza sativa* | NP_001050506.1 | 404 |
|  | HveIF4AIII | *Hordeum vulgare* | BAJ95254.1 | 412 |
|  | ZmeIF4aIII a | *Zea mays* | NP_001105848.1 | 407 |
|  | ZmeIF4aIII b | *Zea mays* | XP_008646449.1 | 407 |
|  | SbeIF4AIII | *Sorghum bicolor* | XP_002454843.1 | 407 |
|  | BdeIF4AIII | *Brachypodium distachyon* | XP_003570724.1 | 410 |
|  | OseIF4AI | *Oryza sativa* | NP_001046321.2 | 416 |
|  | OseIF4AII a | *Oryza sativa* | NP_001058481.1 | 414 |
|  | OseIF4AII b | *Oryza sativa* | NP_001045878.1 | 414 |
| Dicots | AteIF4aIII a | *Arabidopsis thaliana* | NP_188610.1 | 408 |
|  | AteIF4aIII b | *Arabidopsis thaliana* | NP_175549.1 | 392 |
|  | GmeIF4aIII a | *Glycine max* | XP_003533706.1 | 407 |
|  | GmeIF4aIII b | *Glycine max* | XP_003546395.1 | 406 |
|  | MteIF4aIII | *Medicago truncatula* | AFK38550.1 | 406 |
|  | VveIF4aIII a | *Vitis vinifera* | XP_002275011.1 | 412 |
|  | VveIF4aIII b | *Vitis vinifera* | CAN62124.1 | 398 |
|  | SteIF4aIII | *Solanum tuberosum* | XP_006366335.1 | 409 |
|  | AteIF4aI | *Arabidopsis thaliana* | NP_566469.1 | 412 |
|  | AteIF4aII a | *Arabidopsis thaliana* | NP_175829.1 | 412 |
|  | AteIF4aII b | *Arabidopsis thaliana* | NP_177417.1 | 414 |
| Green Algae | CreIF4AIII | *Chlamydomonas reinhardtii* | XP_001699375.1 | 392 |
|  | OteIF4AIII | *Ostreococcus tauri* | XP_003082452.1 | 404 |
| Yeast | Fal1p | *Saccharomyces cerevisiae* | NP_010304.3 | 399 |
|  | Tif2p | *Saccharomyces cerevisiae* | NP_012397.1 | 395 |
| Vertebrates and Invertebrates | HseIF4AIII a | *Homo sapiens* | NP_055555.1 | 411 |
|  | MmeIF4AIII a | *Mus musculus* | NP_619610.1 | 411 |
|  | MmeIF4AIII b | *Mus musculus* | BAC36054.1 | 411 |
|  | DmeIF4AIII a | *Drosophila melanogaster* | NP_649788.2 | 399 |
|  | DmeIF4AIII b | *Drosophila melanogaster* | AAL90373.1 | 399 |
|  | XteIF4AIII a | *Xenopus tropicalis* | B7ZTW1.1 | 415 |
|  | XteIF4AIII b | *Xenopus tropicalis* | NP_001107349.1 | 415 |
|  | DreIF4AIII | *Danio rerio* | NP_957372.1 | 406 |
|  | HseIF4AI a | *Homo sapiens* | NP_001407.1 | 406 |
|  | HseIF4AI b | *Homo sapiens* | NP_001191439.1 | 347 |
|  | HseIF4AII | *Homo sapiens* | AAH15842.1 | 407 |
