# Supplementary material for: Two highly similar DEAD box proteins, OsRH2 and OsRH34, homologous to eukaryotic initiation factor 4AIII, play roles of the exon junction complex in regulating growth and development in rice
Source: BMC Plant Biol. 2016 Apr 12;16:84. doi: 10.1186/s12870-016-0769-5 (PMC4830029; doi:10.1186/s12870-016-0769-5)
Supplement: Additional file 3: — BiFC analysis of the interaction between OsRH2 and OsRH34. (PPTX 122 kb) [file 12870_2016_769_MOESM3_ESM.pptx]

## Slide 1
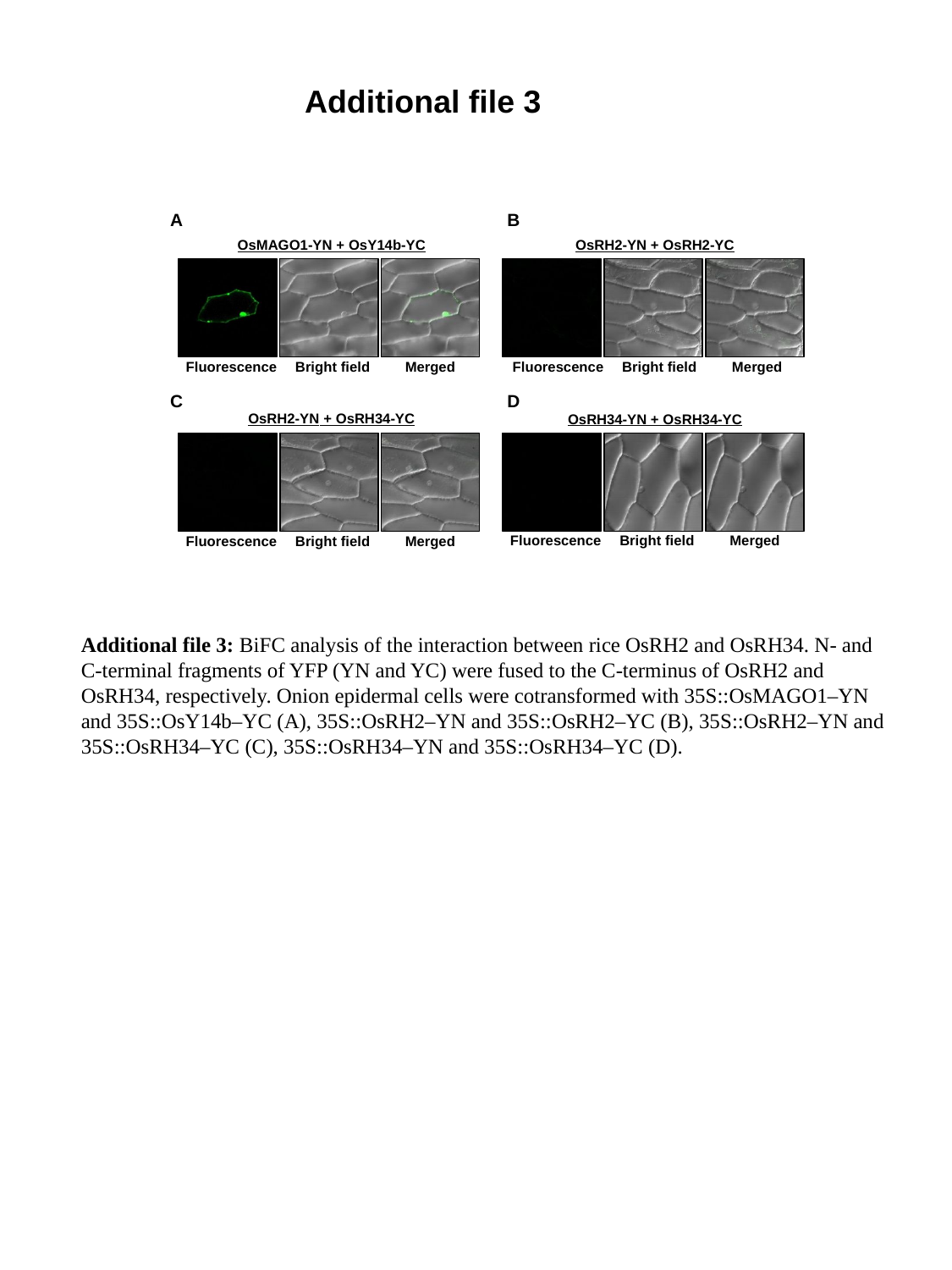

Additional file 3
A
B
OsMAGO1-YN + OsY14b-YC
Fluorescence
Bright field
Merged
OsRH2-YN + OsRH34-YC
Fluorescence
Bright field
Merged
OsRH2-YN + OsRH2-YC
Fluorescence
Bright field
Merged
OsRH34-YN + OsRH34-YC
Fluorescence
Bright field
Merged
C
D
Additional file 3: BiFC analysis of the interaction between rice OsRH2 and OsRH34. N- and C-terminal fragments of YFP (YN and YC) were fused to the C-terminus of OsRH2 and OsRH34, respectively. Onion epidermal cells were cotransformed with 35S::OsMAGO1–YN and 35S::OsY14b–YC (A), 35S::OsRH2–YN and 35S::OsRH2–YC (B), 35S::OsRH2–YN and 35S::OsRH34–YC (C), 35S::OsRH34–YN and 35S::OsRH34–YC (D).
